# Structure and dynamics of motor-driven microtubule bundles†

**DOI:** 10.1039/d3sm01336g

**Published:** 2024-06-14

**Authors:** Bezia Lemma, Linnea M. Lemma, Stephanie C. Ems-McClung, Claire E. Walczak, Zvonimir Dogic, Daniel J. Needleman

**Affiliations:** a Physics Department, Harvard University Cambridge MA 02138 USA; b Physics Department, Brandeis University Waltham MA 02453 USA dl0346@princeton.edu linnealemma@princeton.edu; c Physics Department, University of California Santa Barbara CA 93106 USA; d Medical Sciences, Indiana University School of Medicine Bloomington IN 47405 USA; e Biomolecular Science & Engineering Department, University of California Santa Barbara CA 93106 USA; f John A. Paulson School of Engineering and Applied Sciences, Harvard University Cambridge MA 02138 USA; g Molecular & Cellular Biology Department, Harvard University Cambridge MA 02138 USA; h Center for Computational Biology, Flatiron Institute New York NY 10010 USA

## Abstract

Connecting the large-scale emergent behaviors of active cytoskeletal materials to the microscopic properties of their constituents is a challenge due to a lack of data on the multiscale dynamics and structure of such systems. We approach this problem by studying the impact of depletion attraction on bundles of microtubules and kinesin-14 molecular motors. For all depletant concentrations, kinesin-14 bundles generate comparable extensile dynamics. However, this invariable mesoscopic behavior masks the transition in the microscopic motion of microtubules. Specifically, with increasing attraction, we observe a transition from bi-directional sliding with extension to pure extension with no sliding. Small-angle X-ray scattering shows that the transition in microtubule dynamics is concurrent with a structural rearrangement of microtubules from an open hexagonal to a compressed rectangular lattice. These results demonstrate that bundles of microtubules and molecular motors can display the same mesoscopic extensile behaviors despite having different internal structures and microscopic dynamics. They provide essential information for developing multiscale models of active matter.

## Abstract 2

Microscopic structure tuned by depletant concentration dictates mesoscale dynamics in extensile kinesin-driven microtubule bundles.

## Graphical abstract

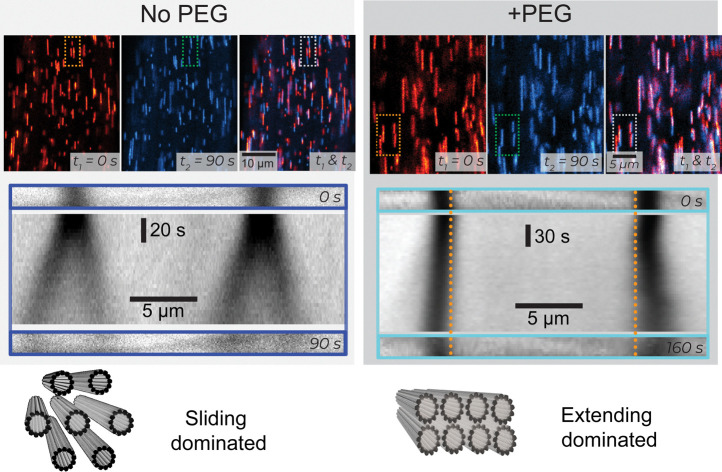

## Introduction

Force-generating molecular motors drive the cellular cytoskeleton away from equilibrium, thus enabling diverse life-sustaining processes such as cell division and motility.^1–3^ Creating synthetic materials with similar lifelike capabilities is a grand challenge. A promising route toward this goal is focused on reconstituting soft materials from the bottom up using purified cytoskeletal components. Such efforts revealed diverse self-organized dynamics ranging from aster formation, bulk contractions, flocking transitions, active nematic liquid crystals, spontaneously flowing active fluids, reconfiguring liquid interfaces and droplets, and the formation of tissue-like active foams.^4–11^ Many aspects of these phenomena can be explained by hydrodynamic models, where the force-generating motors are accounted for by coarse-grained active stresses with *ad hoc* postulated symmetries and magnitudes.^12,13^ It remains less understood how the magnitude and the symmetries of the active stresses depend on the underlying structure and dynamics of the microscopic units from which these systems are composed.^14–22^

Developing predictive multiscale models relating macroscopic active stresses to microscopic behaviors requires bridging multiple length scales and time scales. For example, nanometer-sized molecular motors can drive self-organized turbulent flows with millimeter-scale dynamics.^6^ Similarly, molecular motors step on millisecond timescales, while those of self-organizing flows can be minutes or hours. A way forward is identifying and characterizing mesoscopic force-generating units that bridge the micro and macro length scales. One example is motor-driven filament bundles, a versatile mesoscopic building block for assembling diverse biological and active matter systems. Within a bundle, molecular motors simultaneously bind to and move along multiple filaments, inducing their relative motion and generating active stresses. However, predicting even simple qualitative features of active bundles, such as whether they will contract or expand, remains challenging.^6,14,22^

Experiments on extensile microtubule bundles have revealed two classes of microscopic filament dynamics. (1) In two-dimensional active nematics formed with kinesin-1 clusters and a depletion agent, the velocity characterizing the separation between a microtubule pair displayed an unimodal distribution and increased linearly with increasing filament separation, indicating telescoping extension.^23^ (2) In microtubule bundles formed with kinesin-14 and no depletion agent, microtubules displayed a bi-modal distribution of velocities, with antiparallel populations of microtubules moving apart at a constant speed that was independent of filament separation.^19^ These two systems are different in several aspects including the experimental geometry, the presence or absence of a depletion agent, and the identity of molecular motors. Kinesin-1 clusters have multiple motor domains, while kinesin-14 has a motor domain and a passive binding domain. It is unclear which of these factors determines the mesoscopic bundle dynamics.

We study microtubule bundles driven by kinesin-14 motors with varying concentrations of depletion agents. Increasing the concentration of the depletion agent induced a transition in the microscopic filament motions between the above-described regimes without affecting the overall extensile bundle dynamics. Small-angle X-ray scattering (SAXS) experiments demonstrated that the transition in the microscopic filament dynamics was concurrent with a structural transition from open hexagonal to tight rectangular packing.

## Experimental methods

We expressed and purified a full-length *Xenopus* kinesin-14 clone XCTK2 from baculovirus-infected Sf-9 cells (Invitrogen).^24^ The active samples consisted of stabilized microtubules mixed with 0.54 μM kinesin-14, 80 mM PIPES (piperazine-*N*,*N*′-bis), 5 mM MgCl_2_, 1 mM EGTA, with a pH of 6.8 adjusted with potassium hydroxide. The reaction contained 1.4 mM ATP (Adenosine triphosphate), which kinesin-14 hydrolyzes to ADP (Adenosine diphosphate) to step along a microtubule. In addition, we added 0.034% pyruvate kinase (PK/LDH, Sigma P-0294) and 52 mM PEP (Phosphoenolpyruvate, Beantown Chemicals 129745), which phosphorylates ADP (Adenosine diphosphate) to ATP, thus maintaining constant kinesin dynamics. To suppress photobleaching, we added 4.2 mM DTT (dithiothreitol, ACROS Organics 16568), 2.5 mg mL^−1^ glucose (Sigma G7528), 0.03 mg mL^−1^ catalase (Sigma C40), and 0.17 mg mL^−1^ glucose oxidase (Sigma G2133). When noted, experiments included 35 kDa polyethylene glycol (PEG).

Tubulin was purified from bovine brains.^25^ It was polymerized and stabilized into microtubules by mixing 60 μM tubulin with 3 mM of the non-hydrolyzable GTP analog GMPcPP (guanosine-5′-[(α,β)-methyleno]triphosphate, Jena Biosciences NU-405), and a solution of 1 mM DTT, 80 mM PIPES, 2 mM MgCl_2_, 1 mM EGTA in DI water adjusted to a pH of 6.8 with KOH. For imaging, we labeled 3% of tubulin monomers with a fluorescent dye, NHS-Alexa-Fluor 647 (Invitrogen, A-20006), by a succinimidyl ester linker.^26^ The solution was incubated in a water bath at 310 K for one hour and then left at room temperature for 6 hours. Polymerized microtubules were flash-frozen in liquid nitrogen and were subsequently thawed before sample preparation. Motions of individual filaments were visualized using tracer microtubules. We labeled tubulin with NHS-Azide using the same protocol as NHS-Alexa-Fluor 647 and subsequently used click chemistry to label microtubules with DBCO-Alexa-Fluor 488.^27^ Samples were mixed such that 1 in 10 000 microtubules were labeled in this manner. Microtubule polydispersity was previously measured as a log-normal distribution with mean 4.9 μm, mode 2.8 μm.^10^

Imaging flow chambers consisted of a glass top and bottom, with parafilm spacers, with dimensions of 1.5 mm × 0.1 mm × 18 mm unless noted otherwise. They were sealed with NOA 81 UV Adhesive (Norland Products, 8101) at both ends. The glass was coated with a polyacrylamide brush to suppress proteins' adsorption.^27,28^ To bond the parafilm spacers to the glass, we warmed the parafilm and glass sandwich to 338 K and pressed them onto the glass with the rounded end of a PCR tube. This process led to chambers that were 80–100 μm in height.

We used a Nikon Ti2 microscope with an Andor Zyla sCMOS camera and a 4× Nikon Plan Apo Lambda (NA, 0.2) objective for low magnification data on the macroscopic behavior of the bundles or a 40×/1.15NA Nikon Apo LWD WI objective for high magnification data on the microscopic behavior of the bundles. We used a Leica SP8 Confocal with a 20×/0.75NA objective for confocal imaging and photobleaching experiments. We simultaneously imaged Alexa-647 labeled microtubules and unbleached Azide-DBCO-488 labeled tracer microtubules. When using the SP8 laser, bleaching was most efficient at low magnifications (*M*) and high NA since *I* ≈ NA^4^/*M*^2^. To bleach bundles, we used a 633 nm laser to scan two thin lines ∼15 μm apart for 5 seconds at maximum laser intensity. The short duration of the bleached signal minimized the initial blurring of the bleach mark due to microtubule movement. Bleaching of bundles was conducted along the fast axis of the confocal scan head, coinciding with the longitudinal axis of the microfluidic chamber. Consequently, all imaged and analyzed bundles were aligned with the transverse axis of the microfluidic chamber. Furthermore, this analysis was restricted to bundles that exhibited minimal movement out of the confocal field of view, ensuring consistent observation conditions. Custom Matlab code was used to analyze bleach split behavior.^29^

For Small-angle X-ray scattering, we used a XENOCS Genix 50W X-ray microsource with a wavelength of 1.54 Å and an EIGER R 1M detector. The sample to detector distance was 3.4 m, and images contain a *Q* range of 0.075 nm^−1^ to 2.185 nm^−1^. We used Silver Behenate as a calibration standard. We loaded samples into 1.5 mm Quartz Capillary Tubes (Hilgenberg #4017515) with 0.01 mm thick walls and then centrifuged the liquid to the bottom of the capillary by physically swinging the capillary tube. We sealed the capillaries using NOA 81 UV Adhesive (Norland Products, 8101). We loaded multiple capillary tubes onto a holder in front of a SAXS diffractometer. We aligned samples with the X-ray path near the bottom of the capillary. The detector integrated the X-ray scattering signal for 10 minutes, once an hour, for 12 hours unless otherwise noted. Samples were checked by microscope to ensure the sample was active before and after the experiment (Video 1, ESI†). X-ray scattering data consisted of two camera positions with an overlap. This multi-position acquisition allowed for the integration of signal at higher *q*. We masked and radially averaged the integrated scattering data using the Nika package for Igor Pro.^30^ The SAXS data shown, unless otherwise stated, is the average scattering curve over 12 hours of acquisition.

We measured the scattering due to tubulin and microtubules without kinesin. *I*(*q*)_tubulin_ of unpolymerized tubulin showed a decay with no resolvable peaks (Fig. S2A, ESI†). *I*(*q*)_MT_ due to polymerized microtubules displayed broad peaks at *q* ≈ 0.028 Å^−1^ and ≈ 0.055 Å^−1^. For instance, *L* = 2π/(0.028 Å^−1^) ≈ 22 nm, which is roughly the diameter of a microtubule.

The experimental scattering *I*(*q*) of microtubules after subtracting the background scattering due to free tubulin *I*(*q*)_tubulin_ revealed the form factor *F̂*(*q*) of microtubules with no higher-order positional structure (Fig. S2B, ESI†). We then performed SAXS experiments at various PEG concentrations with and without kinesin-14. We observed similar scattering curves at high PEG with and without kinesin but significantly different scattering curves at low PEG with and without kinesin (Fig. 4A and Fig. S2C, ESI†).

## Results

### Extensile dynamics of microtubule bundles

**Fig. 1:**
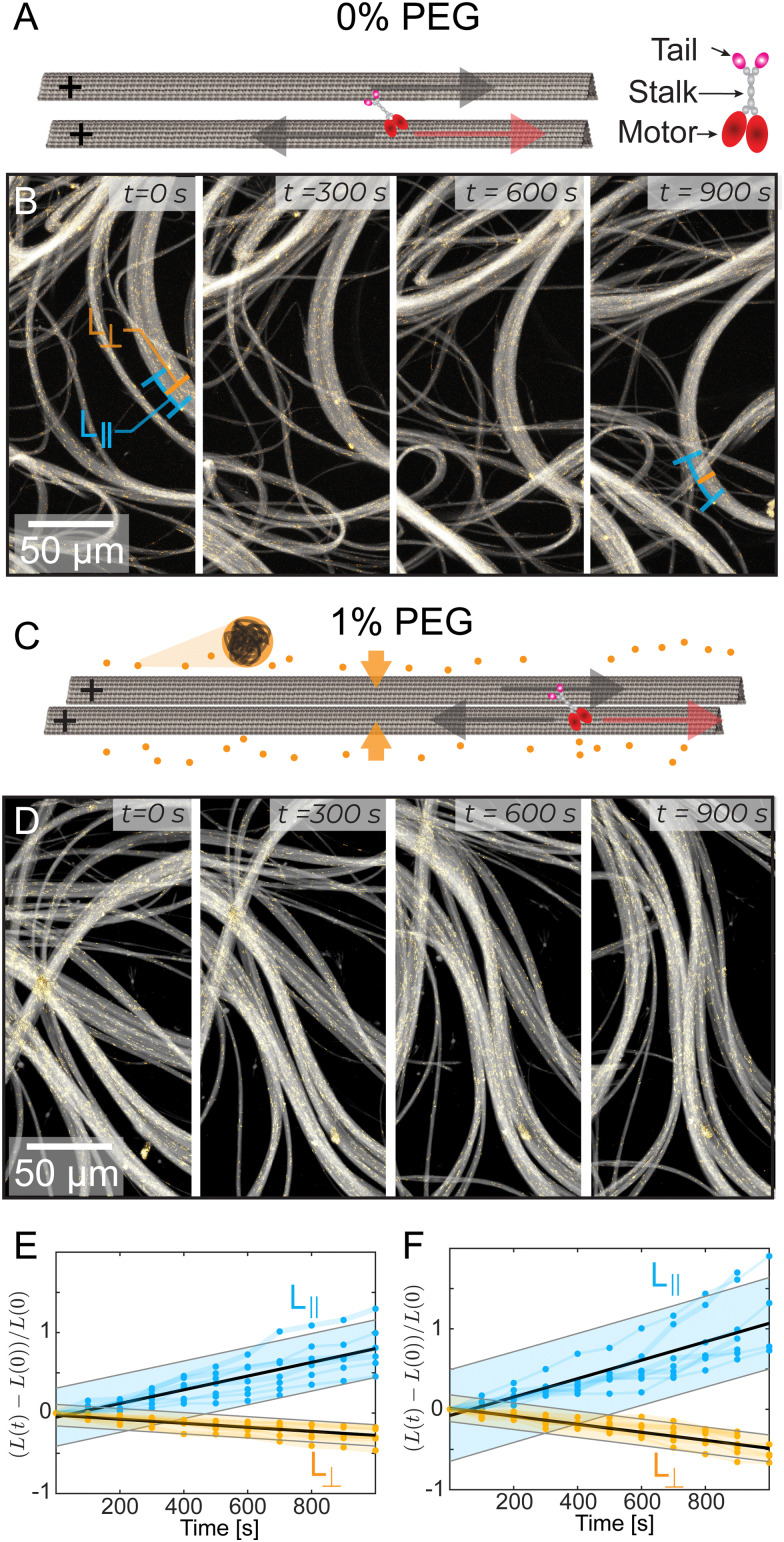
Microtubules, driven by kinesin-14, bundle and display macroscopic extensile dynamics with and without a depletion agent (PEG). (A) and (C) A red arrow indicates kinesin motion and a grey arrow indicates microtubule motion. The kinesin-14 bundles the microtubules with the tail domains attached to one microtubule and the motor domains attached to the other microtubule. In (C), the addition of 1% PEG causes a depletion interaction, further bundling the microtubules. (B) and (D) Time sequence of kinesin-14/microtubule bundles extending and buckling with yellow tracer microtubules, (B) without PEG, and (D) with 1% PEG. The lines at 0s and 900s in (B) demonstrate measurements of *L*_||_ and *L*_⊥_ respectively. (E) and (F) The length *L*_||_ along the bundle or width *L*_⊥_ perpendicular to the bundle, normalized by the initial bundle dimensions *L*_o_ (*N* = 7). Black line represents a linear fit. Shaded bar represents a 95% prediction interval. At 0% PEG (E), *γ*_‖_ = 8.5 × 10^−4^ s^−1^ and *γ*_⊥_ = −2.5 × 10^−4^ s^−1^. At 1% PEG (G), *γ*_‖_ = 1.1 × 10^−3^ s^−1^ and *γ*_⊥_ = −5.0 × 10^−4^ s^−1^.

We studied kinesin-14 microtubule bundles. Kinesin-14 is a dimeric protein with a minus-end directed motor domain at its C-terminus and a passive microtubule-binding domain at its N-terminus (Fig. S1, ESI†).^24,31^ Thus, kinesin-14 both crosslinks microtubules and induces their relative motion.^32,33^ In the presence of kinesin-14, GMPcPP stabilized microtubules coarsened into bundles (Fig. 1A–D). These bundles continuously extend, buckle, and anneal (Fig. 1C, D and Video 2, ESI†). Similar dynamics were previously observed in kinesin-4 and various kinesin-1 active systems.^10,34^ Importantly, kinesin-1 and kinesin-4 systems require a depletion agent that induces an effective microtubule attraction.^35–37^ In comparison, kinesin-14 can generate microtubule bundles both in the absence (Fig. 1A and B) and presence (Fig. 1C and D) of depletion agent.

Quantifying the dynamics of extending bundles has several challenges that stem from the hierarchical motion of the material. At the smallest scales kinesin-14 drives interfilament sliding in microtubule bundles. Such sliding motion powers mesoscopic bundle extension and buckling, which leads to macroscale rearrangement of the entire material. To determine how bundles evolve, we measured their thickness perpendicular to the alignment axis, *L*_⊥_ (Fig. 1B). Additionally, we estimated bundle extension by tracking distinct objects trapped within the bundle, such as collections of tracer microtubules or fluorescent tubulin aggregates separated by distances of 10–50 μm. This revealed how the distance between fiduciary points along the bundle, *L*_||_, changed over time. We found that bundles extend along their length, *L*_||_(*t*), and contract along their width, *L*_⊥_(*t*). Such dynamics are largely independent of PEG concentration (Fig. 1E and F).

### Mesoscale bundle dynamics from photobleaching

**Fig. 2:**
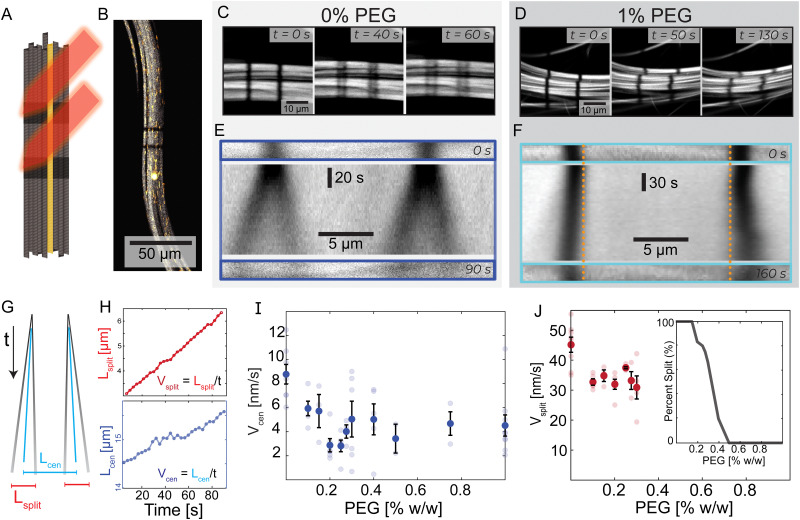
Transition from splitting to non-splitting dynamics. (A) Experimental schematic where a laser bleaches two lines of Alexa-647 labeled microtubule bundles. Alexa-488 labeled tracer microtubules remain fluorescent. (B) A microtubule bundle with tracer microtubules and photobleached lines. (C) and (D) Evolution of bleached lines in PEG-free and 1% PEG samples. PEG-free, two bleached stripes split into two and move in opposite directions. With the addition of PEG, this phenomenon is not present. (E) and (F) Top: Initial bleach lines. Middle: A space-time image showing the temporal evolution of the bleached lines. Bottom: Bleached patterns at *t* = 90 s and *t* = 160 s. Dotted vertical orange lines are a visual reference for the extending bleach marks. (G) A schematic space-time diagram describing bleach lines during splitting dynamics. The black lines indicate the bleach mark trajectories. Blue lines represent the center of mass of each bleach mark. *L*_cen_ is the distance between the two centers of masses. *L*_split_ is the average distance between the two splitting bands. If the lines do not split, *L*_split_ = 0. (H) *L*_cen_ and *L*_split_ increase linearly with time at 0% PEG; *V*_cen_ and *V*_split_ are determined by the respective slopes. Graphs are shifted from *y* = 0 for illustrative purposes. For full graphs, see ESI† (Fig. S3). (I) *V*_cen_ as a function of PEG. Faded dots are individual samples; error bars indicate standard error. (J) *V*_split_, bars show standard error, and faded dots represent individual samples. Inset: The fraction of bundles that display splitting behavior as a function of depletion agent % PEG.

To address the difficulties of measuring the bundle dynamics, we photobleached two stripes within an individual bundle. The two stripes were perpendicular to the bundle's long axis and were several microns apart (Fig. 2A, B and Video 3, ESI†). In 0% PEG samples, each bleached stripe split into two lines moving in opposite directions along the bundle's long axis (Fig. 2C). In contrast, for 1% PEG samples each bleached stripe broadened but did not split (Fig. 2D).

We quantified the dynamics of the bleached patterns in the two regimes (Fig. 2E and F). *L*_cen_ is the distance between the center of masses of the two initially separated bleached marks. If the bleach mark splits, we also measure *L*_split_, the distance between the two peaks that emerged from each initial bleached mark (Fig. 2G). For the splitting scenario, we calculated the center of mass by weighing the position of the two splitting bands *L*_1_ and *L*_2_ by their intensity *I*_1_ and *I*_2_ such that 
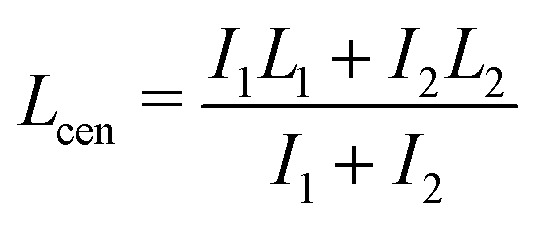
.^19^ Both *L*_split_ and *L*_cen_ increased linearly in time (Fig. 2H, top). Thus, we calculated *V*_split_, which is the speed at which microtubules in each marked region slide apart from each other, and *V*_cen_, which is the overall extension speed of the bundle between the two marked regions for a given lateral spacing (Fig. 2H, bottom). If splitting does not occur, a center of mass can still be calculated, but variables related to splitting are undefined (ESI†).

To quantify the transition between the splitting and blurring regimes, we made samples with a range of PEG concentrations (Fig. S3, ESI†). For all samples, we measured *L*_cen_, and when possible, *L*_split_. The average extensile speed of the system *V*_cen_ gradually decreased from ∼8.8 nm s^−1^ at 0% PEG to ∼4.5 nm s^−1^ at 1% PEG (Fig. 2I). The splitting speed *V*_split_ ranged between ∼45 nm s^−1^ at 0% PEG and ∼30 nm s^−1^ at 0.3% PEG (Fig. 2J). Between 0.15% and 0.4% PEG, only a fraction of microtubule bundles within a sample displayed splitting of the bleached lines. At 0.3% PEG, roughly 2/3 of bundles had splitting dynamics, while at 0.4% PEG, only 1/5 of bundles had splitting dynamics (Fig. 2J, inset). Some bleach lines partially split at these intermediate PEG concentrations while leaving behind a third fainter line, suggesting a coexistence of the two dynamic regimes (Video 3, ESI†).

To quantify the transition between the splitting and broadening regimes, we varied PEG concentration between 0% PEG and 1% PEG (Fig. S3, ESI†). For all samples, we measured *L*_cen_, and when possible, *L*_split_. With increasing PEG concentration, the average extensile speed of the system *V*_cen_ gradually decreased from ∼8.8 nm s^−1^ to ∼4.5 nm s^−1^ (Fig. 2I). The splitting speed *V*_split_ ranged between ∼45 nm s^−1^ at 0% PEG and ∼30 nm s^−1^ at 0.3% PEG (Fig. 2J). Between 0.15% and 0.4% PEG, only a fraction of bundles displayed splitting of the bleached lines. At 0.3% PEG, 2/3 of bundles had splitting dynamics, while at 0.4% PEG, only 1/5 of bundles had splitting dynamics (Fig. 2J, inset). Some bleach lines partially split at these intermediate PEG concentrations while leaving behind a third fainter line, suggesting a coexistence of the two dynamic regimes (Video 3, ESI†). These photobleaching experiments yield crucial insights into the mesoscopic dynamics of the system. Specifically, we observed a striking splitting of bleach lines within bundles in the low-PEG regime, which was absent in the high-PEG regime. This indicates a coherent antiparallel sliding of microtubules in bundles made with low PEG but not in bundles made with high PEG.

### Microscale filament dynamics from tracer microtubules

**Fig. 3:**
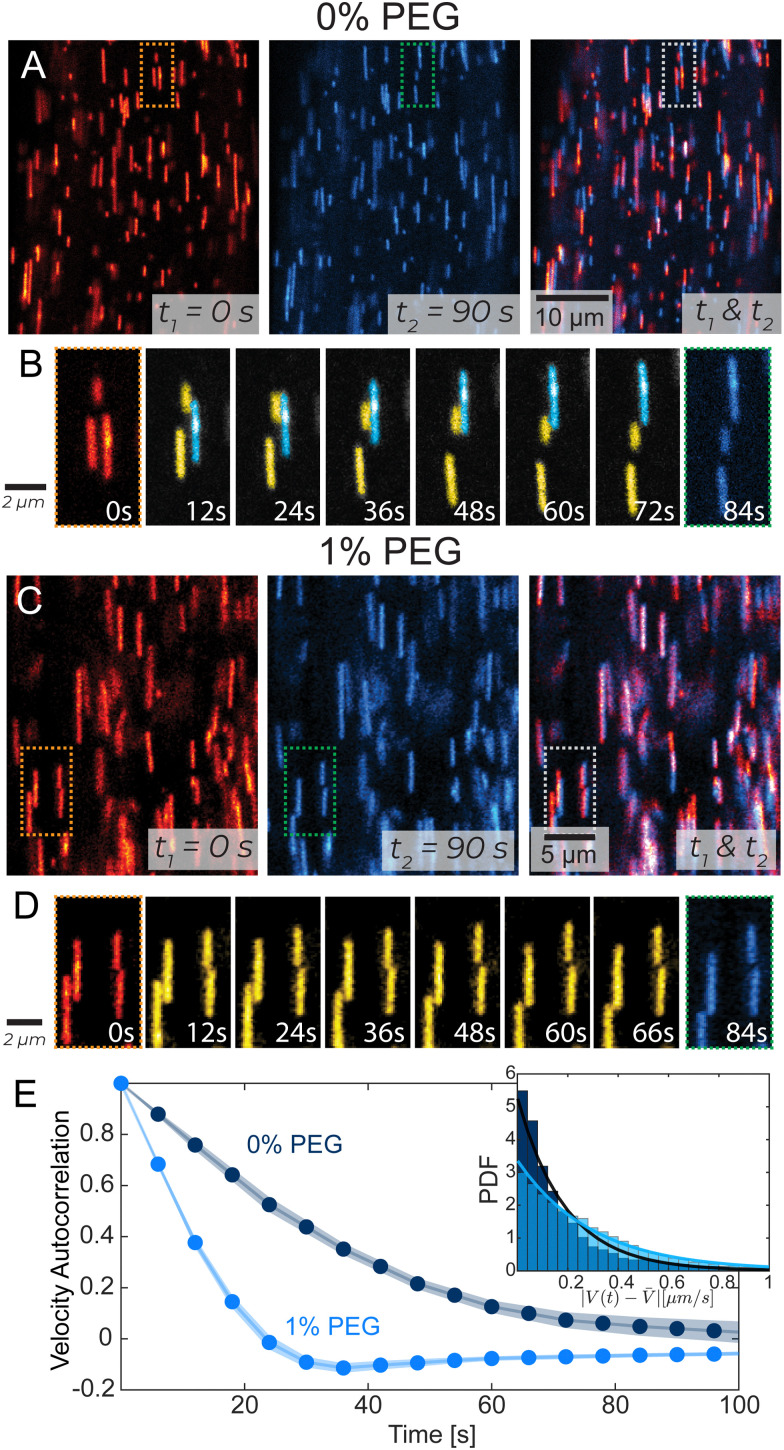
Tracking single microtubules in extensile bundles. (A) and (C) Tracer microtubules within a bundle. The initial frame is in red, and the final is in blue. The overlay shows the net movement of the microtubules over 90 s. (B) and (D) In PEG-free bundles, microtubules exhibit continuous motion with distinct populations of persistently up-moving (cyan) and down-moving (yellow) microtubules. For 1% PEG samples tracer microtubules exhibit more frequent changes in their speed and direction. Microtubule dynamics correspond to dotted regions in (A) and (C). (E) Velocity–velocity temporal autocorrelation of tracer microtubules. Shaded error bars indicate a 95% confidence interval. Inset: Distribution of the difference between the microtubule velocity and the average microtubule velocity |*V*(*t*) − *V̄*|. Lines indicate exponential fits with exponents of 0.19 s μm^−1^ and 0.29 s μm^−1^ for 0% PEG and 1% PEG, respectively.

To visualize individual filament motion, we studied kinesin-14 bundles with 1 in 10 000 labeled microtubules (Fig. 3A, C and Video 4, ESI†). Within a PEG-free bundle, individual microtubules exhibited sustained motion along the bundle's long axis. In 1% PEG samples, individual microtubules exhibited different dynamics, characterized by larger and more frequent changes in both the magnitude and direction of the velocity. These differences are illustrated by the temporal autocorrelation (Fig. 3E). At low PEG concentrations, velocities were correlated on longer time scales. In high PEG samples, correlation decayed much faster, becoming negative, which indicated a characteristic time when microtubules change direction. Additionally, 1% PEG microtubules had a broad distribution of instantaneous velocities. We quantified the deviation from the microtubules' average velocity, *V*(*t*) − *V̄*, over time and found that the probability distribution function of changes in velocity was narrower in 0% PEG samples compared to 1% PEG (Fig. 3E, inset). The spectra of velocity changes for both 0% and 1% PEG fit exponential curves with the exponent *S*_c_ = 1.19 s μm^−1^ and *S*_c_ = 2.90 s μm^−1^ respectively, representing inverse velocity or slowness. These behaviors are consistent with the bleached data (Fig. 2).

### Small-angle X-ray scattering reveals bundle microstructure

**Fig. 4:**
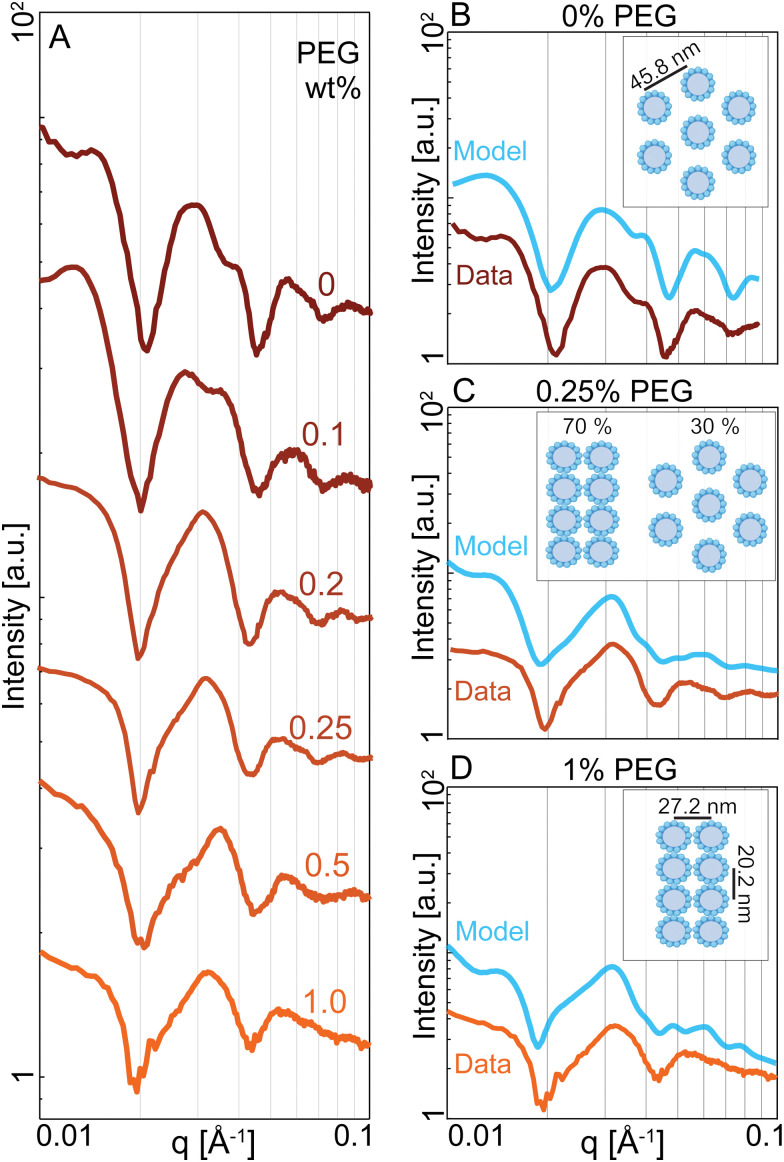
Depletion attraction induces structural transition in microtubule-kinesin-14 bundles. (A) Radially averaged SAXS curves of microtubule bundles with increasing PEG concentration in the presence of kinesin-14. (B) At 0% PEG, the SAXS curve is consistent with a hexagonal lattice of microtubules with a spacing of *L*_h_ = 45.8 nm (cyan). Data is shifted along the *Y*-axis for clarity. (C) At 0.25% PEG, the SAXS curve is consistent with a model that accounts for the coexistence of the hexagonal lattice with a spacing of *L*_h_ = 45.8 nm and a rectangular lattice with *L*_a_ = 20.2 nm and *L*_b_ = 27.2 nm. (D) At 1%PEG, the SAXS curve matches a model of microtubules arranged in a rectangular lattice with lattice parameters *L*_a_ = 20.2 nm and *L*_b_ = 27.2 nm (cyan). The microtubules are deformed and assume an elliptical cross-section. Their major and minor radii are shown in the inset.

We revealed the existence of two distinct regimes of microtubule dynamics, which are determined by varying PEG concentrations. To gain insight into the origin of these different dynamics, we used Small-angle X-ray Scattering (SAXS) to measure the bundle structure (Methods). The scattering curves of microtubule-kinesin-14 bundles were influenced by changing PEG concentration (Fig. 4A). To understand these changes, we modeled various bundle packing configurations using a numerical solver that computes X-ray scattering curves of filamentous structures distributed in random orientations.^38^ We generated several microtubule packing configurations with variable spacing and microtubule numbers (Appendix A). At 0% PEG, the scattering curves were described by a bundle model where microtubules are placed on the hexagonal lattices with a center-to-center spacing of *L*_h_ = 45.8 ± 5 nm (Fig. 4B). In contrast, at 1% PEG, the scattering curves were consistent with a model of microtubules packing in a rectangular lattice (*L*_a_ = 27.2 nm, *L*_b_ = 20.2 nm) (Fig. 4D). This tight rectangular lattice was observed previously for passive microtubule bundles at comparable osmotic pressure of ∼600 Pa.^36,39^ In this limit, the microtubule interior is devoid of the depletant, which causes a differential pressure across the cylindrical shell and the buckling of the microtubule's circular cross-section into an elliptical one. The SAXS curves between the 0% and 1% PEG concentrations could be modeled as a coexistence of the hexagonal and rectangular lattice patterns (Fig. 4C). The scattering model used a small number (<10) of microtubules in both rectangular and hexagonal phases. The broad shape of the peaks and the correspondingly small number of microtubules in the lattice models suggest that the filament packing has short-range order at all concentrations.

## Discussion and conclusions

We showed that kinesin-14-driven microtubule bundles exhibit robust extensional dynamics. Intriguingly, such mesoscale dynamics can be powered by two distinct patterns of microscopic interfilament sliding. Our experiments suggest that different microscopic filament motion is determined by the structural properties of the bundle, which in turn is controlled by the depletant concentration.

**Fig. 5:**
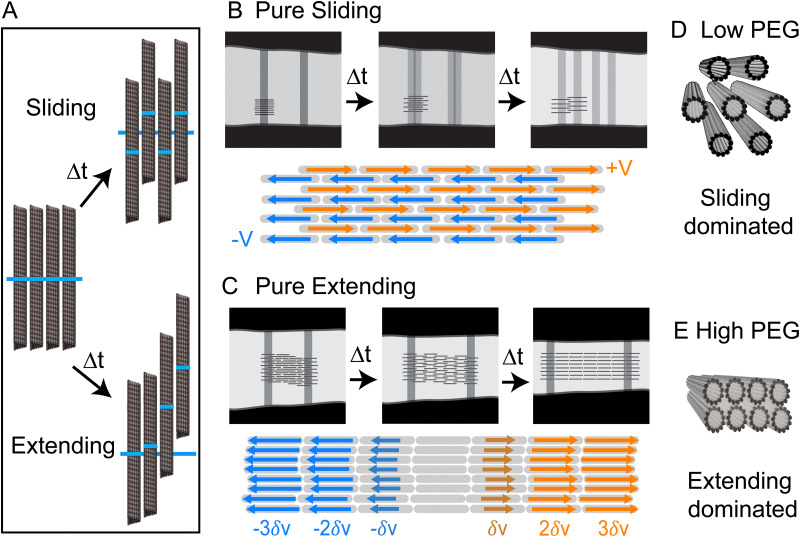
Dynamics of bundles with pure sliding and extension dynamics. (A) The dark blue line shows the initial position of the bleach line. In sliding dynamics filaments marked regions move up or down with a constant velocity. In extensile dynamics, microtubules undergo telescopically amplifying motion. (B) Pure bundle sliding. Half the microtubules move with velocity *V* and half with velocity −*V*. Bleached lines split into two and separate at constant velocities, but the center of mass of the two bleached regions remains at a fixed distance. The bundle thickness remains constant and there is no net bundle extension. (C) Pure bundle extension is generated by a telescoping amplifying motion, which leads to a net bundle extension along its long axis and thinning in the lateral direction. Bleached lines do not split, but their separation increases over time. In this regime, the sliding is highly localized to the neighboring filaments which extend from each other by a small amount. (D) and (E) Hexagonal packing at low PEG concentrations is correlated with predominant sliding dynamics while rectangular packing at high-PEG concentration is correlated with extension dynamics.

In principle, it is possible to envision two types of limiting microscopic microtubule dynamics. In one limit, there are pure sliding dynamics in which filaments move past each other without generating any net extension. In this case, half the filaments move leftward with velocity −*V*, and the other half move rightward with velocity +*V* (Fig. 5A and B). The consequence of such dynamics is that both bleach lines split with *V*_split_ = 2*V*, but their centers of mass remain stationary, resulting in *V*_cen_ = 0. In the other limit, there is a purely *extending* dynamics in which filaments move by extending along each other *via* a telescopically amplifying motion (Fig. 5A and C). In a purely extending bundle, there is no splitting of individual bleached lines, but two bleach lines move apart (*V*_cen_ ≠ 0) as the bundle extends with a velocity that increases exponentially with increasing distance between the bleach lines. Within this conceptual framework, the kinesin-14-bundles transition from a sliding-dominated to an extension-dominated regime. Notably, at low PEG concentrations, we observe predominantly interfilament sliding, yet we also observe net extension with faster overall extension than at higher PEG concentrations. This discrepancy suggests that additional factors, such as an effective intra-bundle friction, influence the net extension speed.

While the SAXS data here revealed structural information about microtubules within a bundle, no experiments have yet ascertained the location of molecular motors within bundles. Nevertheless, the length scales obtained from SAXS experiments allow us to speculate about the possible structural role of molecular motors. AlphaFold predicts that the kinesin-14 motor domain is ∼4 nm. The motor domain is followed by a flexible neck linker so that the entire end-to-end distance is ∼25 nm (Fig. S1, ESI†).^40,41^ At low PEG concentrations, the microtubule bundles have an open hexagonal structure with ∼20 nm distance between two surfaces of the adjacent filaments (Fig. 4C). In contrast, a previous study on microtubules without motors or a depletion agent showed no ordered packing into bundles.^39^ The regular spacing with the kinesin-14 motors suggests that the filaments could be inter-dispersed with motors that are acting to hold the bundle together (Fig. 5D). At higher PEG concentrations, the bundles adopt a rectangular lattice of closely packed microtubules (Fig. 4). Such dense structures may suppress the binding of kinesin-14 from the bundle interior (Fig. 5E). If the kinesin is excluded from the bundle interior, it can only bind to and power the motion of surface microtubules. Alternatively, kinesin binding might occur within the vacancies of the rectangular-packed bundles, but this likely requires the formation of localized filament deformations. Further scattering experiments at higher spatial resolution or within the temporal domain might reveal interesting information about the fluctuations of bundle structure.

The extensile bundle dynamics at higher PEG concentrations are reminiscent of dense 2D active nematics driven by streptavidin-kinesin-1 clusters. The latter showed exponentially increasing velocity along the nematic director.^23^ Comparable exponential extension presumably exists in the kinesin-14 bundles even though the dynamics appeared linear (Fig. 2H). This is likely due to the limited range of measured spatial separations. On short scales, it is challenging to distinguish an exponential from a linear rise. The high density of microtubules in the bundles, particularly in the rectangular packing regime, supports the idea that steric interactions and end collisions could contribute to the active stresses and dynamics of the system.^42^

Large-scale dynamics of cytoskeletal active matter are usually explained using coarse-grained hydrodynamic theories.^13^ In comparison, little is known about the microscopic structure of basic units that power these large-scale non-equilibrium behaviors.^14–20^ Our work provides structural insight into extensile microtubule bundles, a mesoscopic stress-generating motif that powers diverse microtubule-based active matter.^4,10,43^ This work may also be relevant to understanding the dynamics of microtubules in cells where microtubule bundles are present and likely modified by the cellular environment and microtubule-associated proteins. For instance, microtubules in axons are evenly spaced and have roughly hexagonal coordination, while microtubule bundles pillar cells of the inner ear show a striking square packing pattern.^44,45^

Our work represents an initial step toward generating multiscale models of microtubule-based active materials. The detailed microscopic arrangement of kinesin-14 within a microtubule bundle is yet to be elucidated. The generation of a quantifiable axis between pure sliding and pure extending also remains both a theoretical and experimental challenge. These questions must be addressed before a complete multiscale understanding of microtubule-based active matter emerges. Moreover, the multiscale dynamics of other extensile materials have not yet been quantified, so it remains unknown whether the relationship between microscopic structure and mesoscopic dynamics persists in other materials.^10,46–50^ A multiscale understanding will enable the rational creation of active materials with target macroscopic dynamics based on knowledge of the microscopic constituents.

## Data availability statement

The code for D+ SAXS simulations is available at https://github.com/bezlemma under Structure_and_dynamics_of_K14_bundles-2022/D+_LuaScripts/. The code for MATLAB tracking of bleach lines with example stabilized and raw data is available at https://github.com/bezlemma under Structure_and_dynamics_of_K14_bundles-2022/Line_Bleach_MatlabScripts/.

## Conflicts of interest

There are no conflicts to declare.

## Supplementary Material

SM-020-D3SM01336G-s001

SM-020-D3SM01336G-s002

SM-020-D3SM01336G-s003

SM-020-D3SM01336G-s004

SM-020-D3SM01336G-s005

## Appendices

### Appendix

#### (A) Small angle X-ray scattering modelling

We used D+ to model the scattering amplitude of microtubule bundles. D+ is software that computes X-ray scattering curves of basic structures that are isotropically distributed.^38^ First, we modeled a microtubule in the absence of PEG or kinesin. The model assumes a microtubule is a hollow cylinder with 13 protofilaments. Each protofilament is a rod with a diameter of 4.9 nm and a length of 2 μm. The microtubule's center-to-wall radius *r*_MT_ is a free parameter. We compared the numerical model to the experimental scattering *I*(*q*) of microtubules after subtracting the background scattering due to free tubulin *I*(*q*)_tubulin_. Microtubules with a radius of *r*_MT_ = 11.9 nm are consistent with the experiments (Fig. S2A and B, ESI†). We used a solvent electron density of 333 electrons per nm^3^ while the microtubule structures had an electron density of 411 electrons per nm^3^.

We ran simulations of lattice configurations created by repeating patterns of *r*_MT_ = 11.9 nm microtubules. We compared models to the scattering intensity *I*(*q*) from kinesin-14 mediated microtubules in the absence of PEG, again subtracting the measured scattering *I*(*q*)_tubulin_ due to free tubulin. We found that a hexagonal packing of 6 microtubules with a spacing parameter of *L*_h_ = 45.8 nm best fits the data. In contrast, other packing geometries, such as square or rectangular lattices, are incompatible with the experimental SAXS data at low PEG concentrations.

Previous SAXS experiments showed that at high PEG concentration microtubules have elliptical cross-sections and are arranged in a close-packed rectangular lattice.^36,39,51^ Motivated by this information, we created simulations of microtubules with an elliptical cross-section. The microtubule circumference was kept constant by using the Ramanujan simple approximation of an ellipse with the same perimeter as a circle of a given radius, in this case 11.9 nm. We calculated the scattering from microtubules arranged in close-packed rectangular lattices. The rectangular lattices consisted of 1–2 rows of microtubules along the short axis and 3–4 columns of microtubules along the long compressed axis. We included small (<1 nm) variations in microtubule position from an ideal rectangular lattice. The generated scattering curves from an ensemble of these lattices closely match the experimental scattering curves at high PEG concentrations. In these models the microtubule has an elliptical cross-section with a major axis of *r*_b_ = 13.6 nm and a minor axis of *r*_b_ = 10.1 nm, preserving the cross-sectional circumference on an undeformed microtubule. The rectangular packing has a spacing of *L*_a_ = 27.2 nm and *L*_b_ = 20.2 nm. The code to create these geometries is available at https://github.com/bezlemma. The broad shape of the peaks and the correspondingly small number of microtubules in the lattice models suggest that the microtubule packing is less crystalline and more liquid-like with short-range hexagonal or rectangular lattice patterns.
