# Supplementary material for: Structure and dynamics of motor-driven microtubule bundles
Source: Soft Matter. 2024 Jun 14;20(29):5715–23. doi: 10.1039/d3sm01336g (PMC11268426; doi:10.1039/d3sm01336g)
Supplement: SM-020-D3SM01336G-s001 [file SM-020-D3SM01336G-s001.pdf]

Electronic Supplementary Material (ESI) for Soft Matter.
This journal is © The Royal Society of Chemistry 2024

Structure and dynamics of motor-driven microtubule bundles
Supplementary Material

Bezia Lemma, Linnea Lemma, Stephanie C. Ems-McClung, Claire E. Walczak, Zvonimir Dogic and Daniel J. Needleman

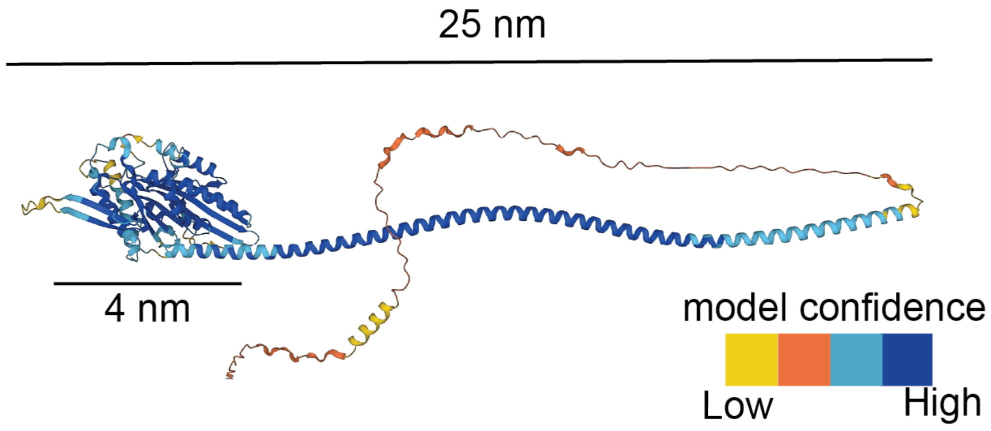

FIG. S1. **AlphaFold prediction of kinesin-14 XCTK2.** The predicted 3D structure of kinesin-14 used in this study. This image is adapted from the uniprot 3D viewer plugin [38,39].

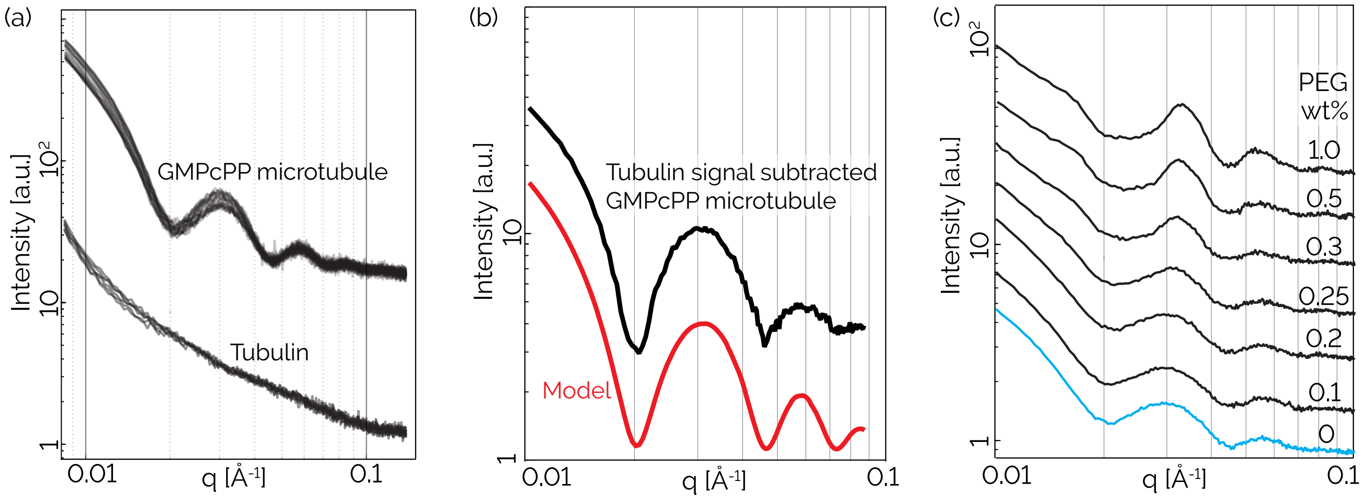

FIG. S2. **Experimental scattering $I(q)$ of GMPcPP microtubules are modeled by a microtubule with r_{MT} = 11.9 nm. (a)** (top) The x-ray scattering curves from samples of GMPcPP polymerized microtubules without kinesin or PEG. (bottom) The x-ray scattering curve from samples of unpolymerized tubulin. **(b)** (top) The average x-ray scattering curve of GMPcPP polymerized microtubules after subtracting the measured unpolymerized tubulin curve. (bottom, red) A numerical scattering curve of a polymerized microtubule calculated by D+, with $r_{\mathrm{MT}} = 11.9$ nm. **(c)** Radially averaged x-ray scattering curves of microtubules without kinesin with increasing concentrations of PEG. All curves shown have been arbitrarily shifted along the Y-axis.

A. Further photobleaching results and bleach line detection criteria

Code for bleach line detection is available at https://github.com/bezlemma, and is based off the code used in S Fürthauer, B Lemma et al, 2019 [17].

Briefly, to create an initial guess at a bleach line's location for an early image in the time course, the 2D data is flattened into 1D along the axis of the bleach line. The maximum bleach intensity $I_{\max}$ and mean intensity along the 1D data set I_{mean} are calculated. We then apply a moving average filter with a width of 5 pixels to minimize noise, and test if there are peaks with a prominence compared to its neighbors greater than $I_{\max}/100$ and a value greater than I_{mean} of the 1D. If there is only one peak that satisfies this criterion no split will be calculated. If there are at least two peaks that satisfy this criteria, the code fits two Gaussian functions to the 1D data set with the location of the two most prominent peaks as the initial guess locations and goodness-of-fit criteria determines if this fit will be used for the remaining images in the dataset.

Below are supplemental graphs of L_{split} and L_{cen} for 6 PEG concentrations with three experiments at each concentration. In splitting bundles, we calculated the center of mass by weighing the position of the two splitting marks L_1 and L_2 by their intensity I_1 and I_2 such that $L_{\text{cen}} = \frac{I_1 L_1 + I_2 L_2}{I_1 + I_2}$. Then L_{split} is the distance between the two peaks L_1 and L_2 which evolve from each bleached mark. Thus non-splitting lines do not have a defined I_2 or L_2. Thus by our definition, L_{split} is not 0, it is undefined.

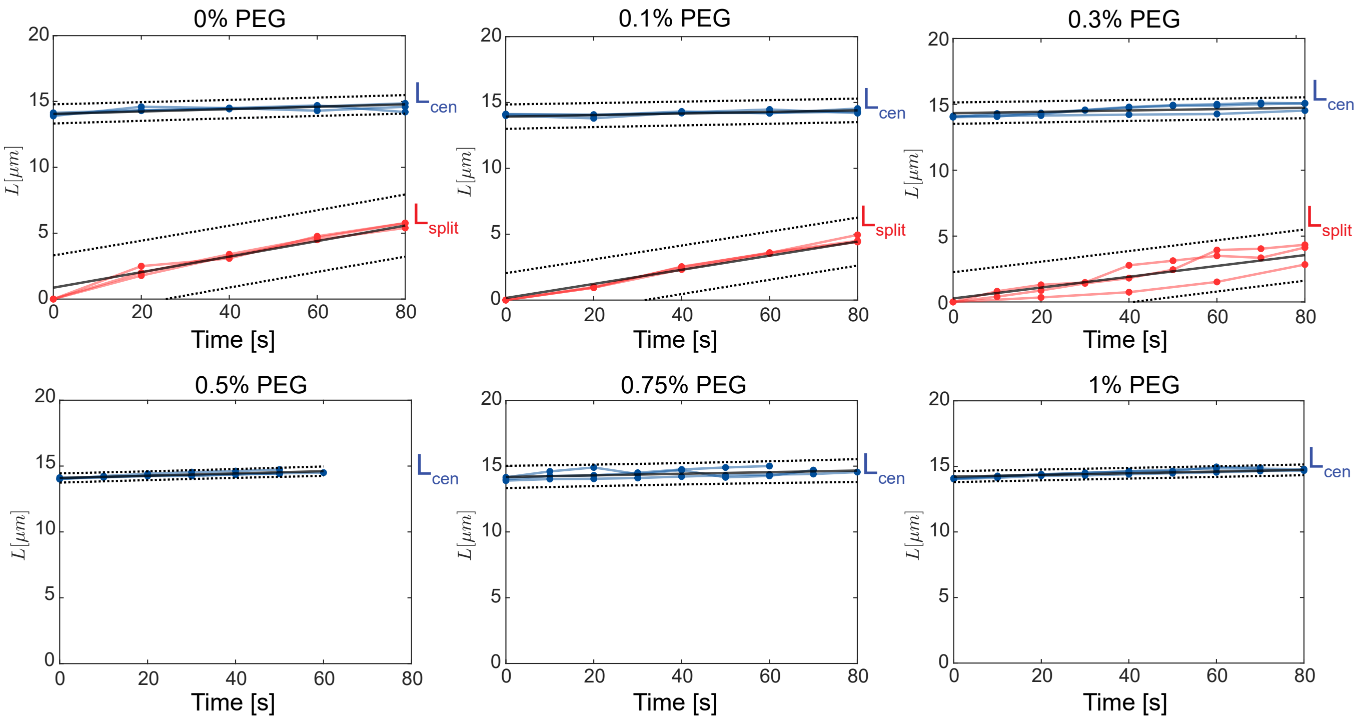

FIG. S3. **L_{split} and L_{cen} time courses for PEG concentrations from 0 to 1%.** The solid black line represents a linear fit, and the dotted black line represents 99% prediction bounds. Above 0.5% PEG line splitting is not detected and thus L_{split} is not defined.

I. SUPPLEMENTARY VIDEOS

- Video 1: Brightfield microscopy of a 1% PEG kinesin-14 sample in a 1.5 mm quartz capillary tube after small-angle x-ray scattering. On the left is a still image of series of samples loaded into capillary tubes and mounted for x-ray scattering.
- Video 2: The macroscopic dynamics of kinesin-14 driven microtubule bundles first without PEG and then with 1% PEG. These dynamics appear qualitatively similar. In grey are fluorescent microtubules, and in yellow are individual tracer microtubules.
- Video 3: A series of bleach line experiments between 0% to 1% PEG in several samples with differently sized bundles. In grey are fluorescent microtubules, which are bleached, and in yellow are individual tracer microtubules, which are not bleached. As PEG concentration increases, the samples transition to non-splitting dynamics. Between 0.2% PEG to 0.5% PEG, bleach lines reveal a coexistence between splitting and non-splitting dynamics.
- Video 4: The motion of tracer microtubules (yellow) within a kinesin-14 driven microtubule bundle (not shown). Without PEG, the tracer microtubules display sliding and extending dynamics. In a sample with 1% PEG, the dynamics of the tracer microtubules are still extending, but exhibit a stuttering motion.
